# Variability in *waxy* (*Wx*) allele, in-vitro starch digestibility, glycemic response and textural behaviour of popular Northern Himalayan rice varieties

**DOI:** 10.1038/s41598-021-91537-0

**Published:** 2021-06-08

**Authors:** Bazila Naseer, H. R. Naik, Syed Zameer Hussain, Asif Bashir Shikari, Nowsheen Noor

**Affiliations:** 1 Division of Food Science and Technology, Sher-e-Kashmir University of Agricultural Sciences and Technology of Kashmir, Shalimar, 190025 India; 2 Department of Food Technology, Islamic University of Science and Technology, Awantipora, 192122 India

**Keywords:** Biotechnology, Nutrition

## Abstract

Eight commonly cultivated and consumed rice varieties of Northern Himalayan regions and a popular high amylose rice variety were characterized at *Wx* locus and evaluated for resistant starch (RS), in-vitro starch digestibility, predicted glycemic index (pGI), glycemic load (GL) and textural parameters. Cytosine and thymine repeats (CT)n at waxy locus (*Wx*) showed high association with apparent amylose content (AAC). Both pGI and GL varied substantially within the selected varieties. The pGI was relatively lower in high and intermediate amylose Indica varieties compared to low amylose Japonica ones. However, *Koshikari* despite being a low amylose variety showed relatively lower pGI and GL, due to its higher RS, dietary fiber, protein and fat content. It was thus presumed that in addition to AAC, RS and other grain components also affect the glycemic response. Inherent resistance to enzymatic hydrolysis was also found to be higher in firm textured and less sticky rice varieties. The genotypes—*Lalat, Basmati-1509* and *Koshikari,* in view of their low to moderate pGI and relatively higher RS content, can be explored in future breeding programmes to develop rice varieties whose consumption will help to prevent hyper/hypo glycemic responses in Northern Himalayan regions, where daily staple diet is rice.

## Introduction

The prevalence of type II diabetes is rapidly increasing in all the parts of the world with most severe implications on the low- and middle-income communities^1,2^. Globally, 463 million people are affected by diabetes, out of which 77 million are reported to be from India^3^. It is expected that by 2030, 578 million adults will be living with diabetes and its management will be one of the biggest health challenges in future.

Rice (*Oryza Sativa* L*.*) is the second most important cereal crop grown in the world and is the staple food to more than 60% of the world population. It holds a significant position in human nutrition. Total global consumption of rice is more than 493.12 million metric tons annually^4^. India is the second largest producer as well as consumer of rice in the world^5^. Rice is a good source of macronutrients especially carbohydrates. However, larger intake of white rice is considered as one of the major reasons for prevalence of type II diabetes in Asian population. Several cohort studies have reported that consumption of more than 300 g/day of white rice increases the risk of type II diabetes by 78%^6^. The association of glycemic index (GI) with increased risk of metabolic syndrome and diabetes is well documented in many previous studies^7,8^. The regular consumption of white rice impairs the glucose metabolism and insulin resistance due to its higher GI^7^.

However, physico-chemical, molecular and genetic make-up determines the digestibility and glycemic response of rice. GI of high amylose rice varieties is usually lower than the waxy ones^9^. Amylose is considered as one of the predominant traits which governs the cooking quality of rice. Amylose content of rice is regulated by *waxy (W*_*x*_) gene that codes for Granule Bound Starch Synthase (GBSS) protein. Simple sequence repeat (SSR) marker RM190 linked to *Wx* phenotype has been reported to explain around 60% of variance for amylose content in rice^10^. The RM190 is based on dinucleotide repeat CT that varies from (CT)_8_ to (CT)_20_ and the marker is located in 5′ Untranslated Region (UTR) of Waxy exon 1^11^. The corresponding length of repeats within SSR fragments are associated with the specific amylose class. Fitzgerald et al.^12^ studied the association between amylose content, *waxy* allele and GI in improved and traditional rice varieties of Asia. They observed strong correlations between amylose content, *waxy* locus and GI across all the samples which indicated that amylose is the major grain constituent that affects the GI. However, resistant starch (RS) which act as a non-glycemic starch fraction in rice also affect its digestibility. Typically, type-5 resistant starch is formed in rice due to interaction of amylose and lipids. Such complexes are composed of hydrophobic polyglucan chains and are responsible for lowering of GI due to their resistance to enzymatic hydrolysis^13^. Amylose–lipid complexes organize into a partially ordered structure typically known as V-type crystalline lattice^14^. Both, amylose and RS are inversely related to GI^15^. Therefore, there is a need to identify specific rice varieties having high amylose and relatively high RS content in different rice consuming regions.

Although, rice is relatively known to deliver a higher glycemic response but consumption of rice is linked to cultural preference and satiety effects among Asian population. Therefore, dietary management of type II diabetes through a staple food like rice holds a viable scope to curb its future escalation. Thus, there is a scope to screen the commonly consumed rice varieties of Northern Highland Himalayan regions for *Wx* allele characterization, in-vitro starch digestibility and glycemic index. To the best of our knowledge, rice varieties grown in Northern Highland Himalayan regions have not been tested so far for *Wx* allele characterization, in-vitro starch digestibility and glycemic index. Thus, the present study was conducted with an aim to characterize eight rice varieties commonly cultivated and consumed in Northern Highland Himalayan regions, at *waxy* locus (*Wx*) using RM190 and to study the association of amylose content with inherent allelic variation, in-vitro starch digestibility, predicted glycemic index, glycemic load and textural parameters in selected rice varieties.

## Material and methods

### Raw materials

Five Indica (*SR-2*, *Basmati-1509*, *China-1007*, *Chenab* and *Jhelum*) and three Japonica (*Koshikari*, *SKUA-402* and *K-332*) paddy varieties commonly grown in Northern highland Himalayan regions were procured from the Mountain Research Centre for Field Crops, Khudwani, Sher-e-Kashmir University of Agricultural Sciences and Technology, Kashmir, (SKUAST-K), India^16^. While as *Lalat*, a high amylose rice variety was procured from Research and Seed Processing Centre, Odisha University of Agriculture and Technology, Bhubaneswar, India. The material was procured from the registered seed centers, in compliance with the institutional guidelines. All the paddy varieties were milled in a modern rice mill (ASR RM 209). Milling percentage recorded for different varieties was 70 ± 5%. Polished head rice grains obtained from each variety were dried to a moisture content of 11–12 ± 0.5% in a cabinet drier (LSI-EC-STB), Lab Solutions, India at a temperature of 40 ± 5 °C^17^. A portion of polished head rice grains were ground in a laboratory mill (Pertin, USA) to obtain rice flour. Both dried polished head rice gains and rice flour were stored in separate air tight containers under ambient conditions for further analysis.

### Determination of apparent amylose content

Standard protocol as per Megazyme assay kit (K-AMYL 06/18) was followed for determination of apparent amylose content (AAC). Rice flour (20 mg) was mixed with 1 mL dimethyl sulphoxide (DMSO) in a falcon tube and stirred on a vortex shaker. The tube was placed in a boiling water bath for 1 min to solubilize the sample. The contents were thoroughly mixed on vortex again and then heated for 15 min in a boiling water bath with intermittent shaking. Ethanol (2 mL, 95%) was added to tubes with continuous stirring. 4 mL of ethanol was added to tubes and mixing was done by inverting the capped tubes. After a resting time of 15 min, tubes were centrifuged at 4000*g* for 5 min. Supernatant was discarded and tubes were dried on a tissue paper. The pellets recovered were mixed with 2 mL of DMSO and shaken gently. The tubes were placed in a boiling water bath for 15 min with intermittent mixing to prevent lump formation. Concanavalin A (Con A) (4 mL of 30% concentrated Con A solvent) was immediately added to tubes with thorough mixing and contents were transferred to 25 mL volumetric flask. The volume was raised to 25 mL by Con A solution and solution was filtered through Whatman filter No. 1. 1 mL of filtered extract was mixed with 0.5 mL of Con A solution in Eppendorf tubes. The tubes were centrifuged after 1 h at 14,000*g* for 10 min. One mL of supernatant was mixed with 3 mL of 100 mM sodium acetate buffer (pH = 4.5). The tubes were heated in boiling water bath for 1 min followed by heating at 40 °C for another 5 min for temperature equilibration. The samples were incubated at 40 °C with 0.1 mL of amyloglucosidase (3300 U/mL)/α-amylase (500 U/mL) enzyme solution for 30 min and centrifuged at 2000*g* for 5 min. Aliquots of 0.1 mL were taken from supernatant and 3 mL of GOPOD reagent was added to each tube. Incubation of tubes were done at 40 °C for 20 min along with reagent blank and d-glucose standard. Absorbance of samples was measured at 510 nm and amylose was calculated from the standard formula given in kit manual.

### Marker analysis of rice genotypes at *Wx* locus

A set of nine rice germplasm lines were validated at *Wx* locus on chromosome 6 with the help of simple sequence repeat (SSR) marker RM190 which is known to be associated with Granule Bound Starch Synthase (GBSS) enzyme that regulates amylose content in rice and exhibits dinucleotide (CT)_n_ repeat polymorphism. The genomic DNA was extracted from young, fresh and healthy 2 weeks old seedlings following CTAB (Cetyl-Tri-Methyl Ammonium Bromide) method as described by Murray and Thompson^18^. Polymerase Chain Reaction (PCR) reaction mix contained ~ 25 ng of DNA, 10× PCR buffer (10 mMTris, pH 8.4, 50 mMKCl, 1.8 mM MgCl_2_), 2 mM dNTPs (Thermo Fisher Scientific, Waltham, USA), 5 pmol each of forward and reverse primer and 3 U of Taq DNA polymerase (Thermo Fisher Scientific, Waltham, USA a) in a reaction volume of 10 μL. After an initial denaturation step of 94 °C for 5 min, the amplification was carried out for 35 cycles comprising 30 s each of 94 °C, 55 °C and 72 °C. The final extension step was performed for 10 min at 72 °C followed by storage at 40 °C. PCR products were resolved through Polyacrylamide Gel Electrophoresis. Non-denaturing polyacrylamide gel (12%) was prepared by dissolving 29 g of acrylamide and 1 g of N,N′-methylene-bis-acrylamide in 0.5× TBE buffer. Afterwards 20% ammonium persulfate (APS) solution was also prepared. Polymerization of gel was affected by addition of 20 µL of tetramethyl-ethylene-diamine (TEMED) and 200 µL fresh 20% APS. About 10 µL of PCR product was loaded into each well of the polyacrylamide gel along with a 100 bp size DNA ladder (Thermo Fisher Scientific, Waltham, USA). And finally the gel was run at a constant voltage of 110 V until the dye reached a defined position, normally for ~ 70 min. 1 L of fresh impregnation solution was prepared by dissolving 1.5 g of AgNO_3_ in 1 L of distilled water followed by preparation of 1 L of fresh development solution by dissolving 10 g of NaOH in 900 mL of distilled water. After adding 1 mL of 37% formaldehyde, the final volume was adjusted to 1 L. After electrophoresis, the polyacrylamide gels were stained using the above silver staining protocol, which unambiguously detected the banding pattern of SSR marker^19^.

### Cooking of rice grains

Head rice grains (5 g) were placed in a beaker containing 100 mL of hot boiling water. Grains were cooked in a boiling water bath (100 ± 2 °C) for 15 min. After 15 min and every minute thereafter, 1–2 grains were examined by pressing between two glass slides to check if any opaque core was left uncooked. All the varieties were cooked in a similar manner till 90% of grains became translucent and were completely cooked^20^. Cooking was completed in about 20 ± 2 min and excess water was drained. Cooked and drained kernels were analyzed for below mentioned parameters.

### Resistant starch

Resistant starch content of cooked rice was measured as per the standard AACC protocol^21^ using Megazyme Assay Kit (Megazyme International, Wicklow, Ireland). 100 mg of homogenized sample was put into falcon tubes ensuring no residue remains adhered to the walls. Four mL of pancreatic alpha-amylase (Sigma-Aldrich, UK) (10 mg/mL in Tris-maleate buffer, pH 6.0) and 300 µL of amyloglucosidase (AMG) (Megazyme, 3300 U/mL) was added to samples and shaken vigorously followed by incubation in a shaking water bath for 16 h at 37 °C. Tubes were removed 16 h latter and absolute ethanol (4 mL) was added to each tube followed by vigorous stirring. Tubes were centrifuged for 10 min at 1500*g* and decanted pellets were re-dissolved in 2 mL and 6 mL of ethanol (50%) in two steps with continuous stirring and centrifugation was repeated again. Potassium hydroxide solution (2 mL, 2 mol/L) was added to tubes and stirred for 25 min in an ice-bath to dissolve the pellets. 8 mL of sodium acetate buffer (1.2 mol/L, pH 3.8) was added to each tube and stirring was done on a magnetic stirrer followed by addition of 0.1 mL of AMG solution (3300 U/mL). The contents were mixed again and incubated at 50 °C in water bath for 30 min. Aliquot of 0.1 mL was taken after centrifugation at 2000*g* for 10 min. 3 mL of Glucose oxidase/peroxidase (GOPOD) reagent [for preparation of GOPOD reagent, glucose oxidase (> 12,000 U), peroxidase (> 650 U) and 4-aminoantipyrine (80 mg) supplied as freeze dried powder was dissolved in 50 mL of GOPOD buffer (p-hydroxybenzoic acid and sodium azide (0.09%w/v)] was added to aliquots in duplicate followed by incubation at 50 °C for 20 min. Absorbance was measured at 510 nm (UV–Vis spectrophotometer, ThermoSpectronic, USA) against reagent blank and RS was calculated from the formula given in the instruction manual.

### In-vitro starch digestibility, predicted glycemic index and glycemic load

*In-vitro* starch digestibility and predicted glycemic index were determined by the standard protocol given by Goñi et al.^22^. All the cooked rice samples (100 mg) were homogenized uniformly for 3 min using Ultra Turrax homogenizer (T25, Ika Labortechnik) to obtain a slurry. These slurry state samples were treated with 10 mL of 1 M HCl–KCl buffer and mixed thoroughly. This was followed by addition of 0.2 mL pepsin solution (1 g prepared in 10 mL of 1 M HCl-KCl buffer, pH = 1.5) and incubation at 40 °C for 60 min in a shaking water bath. Volume was raised to 25 mL by adding 15 mL of 0.1 M tris-maleate buffer (pH = 6.9) followed by stirring and immediately 5 mL α-amylase solution (10 mg/mL in 0.1 M tris-maleate buffer, 2.6 IU) was added to each tube. Time course incubation of enzyme treated samples was done in a shaking water bath at 37 °C for 3 h and aliquot of 1 mL in triplicates were collected after intervals of 0, 30, 60, 90, 120, 150- and 180-min. For estimation of rapidly digestible starch (RDS) and slowly digestible starch (SDS) fractions, additional aliquots (1 mL) collected from the reaction mixture after 20 min (G_20_) and 120 min (G_120_) of incubation time were used for glucose quantification respectively. Enzyme reaction was stopped by immediate boiling to 100 °C for 5 min. Final incubation was carried out at 60 °C for 45 min by adding 1 mL of 0.4 M sodium acetate buffer (pH = 4.75) and 80 µL of AMG solution (3300 U/mL) to each aliquot. Aliquot of 100 µL was used for glucose measurement after centrifugation of tubes at 2000*g* for 10 min. Glucose released after every 30 min was quantified using glucose oxidase peroxidase kit (Megazyme, Ireland). The starch digestion rate was expressed as the percentage of total starch (TS) hydrolyzed after 30 min interval from 0 to 180 min. pGI and hydrolysis index (HI) were calculated using the kinetics of starch digestion. pGI was calculated from the standard formula,

1 $${\text{pGI}} = 39.71 + \left( {0.549 \times {\text{HI}}} \right) .$$

Total starch content was measured using the total starch assay kit (K-TSTA, Megazyme, Bray, Ireland) by standard AACC^21^. 100 mg of powdered sample was mixed with 0.2 mL of 80% ethanol followed by addition of 2 mL cold NaOH solution (1.7 M). The tubes were placed over a magnetic stirrer in an ice-water bath for 15 min. The contents were stirred in between vigorously to avoid any lump formation. 8 mL of sodium acetate buffer (600 mM, pH = 6.8) was added and mixing was done using a vortex shaker. 0.1 mL of each α amylase (3000 U/mL) and AMG (3300 U/mL) were added and mixing was done again for 5 s. Tubes were incubated in a water bath for 30 min at 50 °C. 2 mL of solution was centrifuged at 7000*g* for 5 min. 1 mL of supernatant was mixed with 4 mL of sodium acetate buffer (100 mM, pH = 5.0). After through mixing, 0.1 mL of aliquots were treated with 3 mL of GOPOD reagent and incubated for 20 min at 50 °C. Absorbance was measured at 510 nm and total starch was calculated as g/100 g of sample weight.

Glycemic load of the samples was calculated using the following equation

2 $$GL = \frac{pGI \times available\;carbohydrate\;per\;serving\;size}{{100}}.$$

Standard AOAC^23^ procedures were followed for determination of protein, fat, moisture, ash and crude fiber contents. Total carbohydrate content was determined by subtracting the mean values of protein, fat, moisture, ash and crude fiber content from 100^24^. Dietary fiber was determined using Dietary Fiber system (FIBRA PLUS DF, Pelican equipments, Chennai) by following approved AOAC^23^ procedure. Available carbohydrate content per serving size of 100 g was then calculated as difference of total carbohydrate and dietary fiber^17^.

### Texture profile analysis

Texture profile analysis of cooked rice was carried out by texture analyzer (Stable Micro System, TA-XT2i, Surrey, UK) following the procedure reported by Hussain et al.^25^. Textural parameters like hardness, cohesiveness and stickiness were calculated from the inbuilt Texture Expert software.

### Statistical analysis

One-way analysis of variance (ANOVA) followed by Duncan’s multiple range test was used to determine the statistical difference of measured parameters within the selected rice varieties. Standard statistical software, SPSS was used for data analysis. Pearson’s correlation coefficient was computed to determine the relationships among the analyzed parameters.

## Results and discussion

### Chemical composition

The proximate composition of selected varieties studied previously by authors is given in Table S1^16,17^.

### Apparent amylose content and allelic variation at (CT)_n_ locus

**Figure 1:**
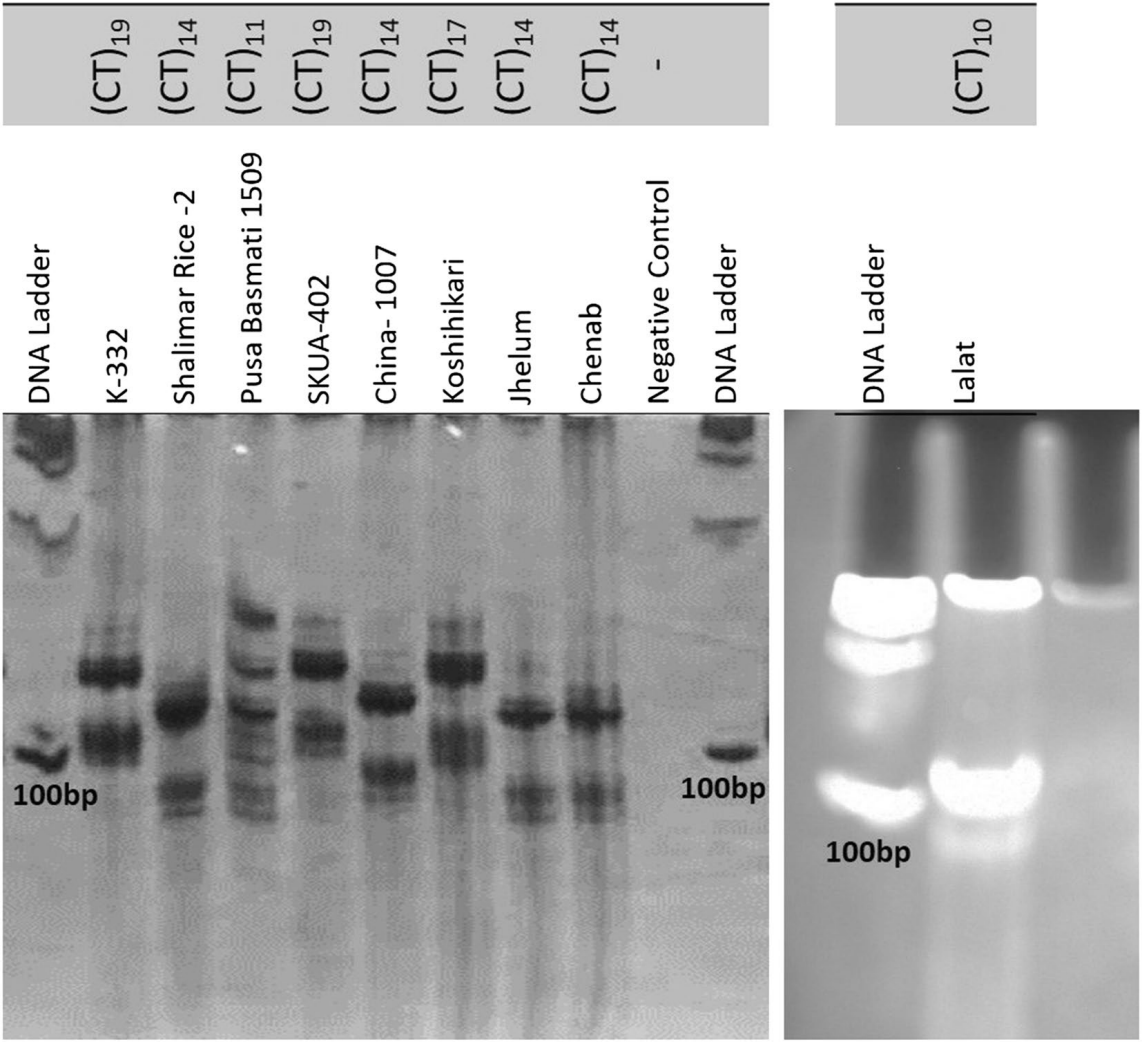
Allelic variation within Waxy gene among rice genotypes at RM190 locus.

Apparent amylose content (AAC) of different rice genotypes varied significantly between 15.40% (*Koshikari-* a short bold variety) to 28.31% (*Lalat—*a long grain variety) (Tables 1 and 2). As per the classification given by Juliano^26^, the Indica varieties namely-*Shalimar Rice-2, Basmati-1509, China-1007, Chenab,* and *Jhelum* were classified as intermediate amylose type, *Lalat* as high amylose variety, whereas Japonica varieties—*SKUA-402, Koshikari* and *K-332* were classified as low amylose type. Feng et al.^27^ also reported lower AAC in Japonica varieties as compared to Indica ones. Amylose content is governed by *Wx* locus that manifests its effect through regulation of starch synthase enzyme GBSS. A gene linked SSR marker RM190 carries dinucleotide (CT)_n_ repeats in the 5′ Untranslated Region (UTR) of Waxy exon 1 of *Wx* gene^11^ and explains the difference between glutinous and non-glutinous rice^28^. Low amylose Japonica rice varieties- *SKUA-402* and *K-332* amplified (CT)_19_ allele, whereas, *Koshikari* amplified (CT)_17_ allele (Fig. 1). Bao et al.^29^ also found the CT_17_ to be the most common waxy microsatellite in glutinous rice accessions. Biselli et al.^28^ also reported that alleles (CT)_17_ to (CT)_19_ represent AAC range of 14.92–23.7%. Among the different Indica varieties, *Lalat*, carried a CT repeat of 10 that is associated with high amylose and *Basmati-1509* amplified (CT)_11_ allele (Fig. 1) which is also associated with high amylose content. However, *Shalimar Rice-2, Jhelum, Chenab* and *China-1107* carried (CT)_14_ allele (Fig. 1) which are associated with low to intermediate amylose content^30^. Cheng et al.^31^ elucidated five *Wx* gene alleles-CT_10_, CT_11_, CT_16_, CT_17_, and CT_18_ among the 15 rice varieties and found that high amylose varieties were associated with shorter repeat alleles, namely CT_10_ and CT_11_ against the low and intermediate amylose varieties carrying longer repeat alleles—CT_16_, CT_17_ and CT_18_. Correlation matrix given in Table 3 also showed highly significant (p < 0.01) negative correlation of AAC with CT repeat (r = − 0.909). Amylose is resistant to enzymatic degradation therefore; high amylose rice varieties usually elicit low GI responses^11^. Overall RM190 is an efficient marker that has been reported to explain 86.4% of variation in AAC^29^. The marker explained the inherent variation of AAC within the rice genotypes selected in the present study as well.

**Table 1:** Salient features of rice (*Oryza sativa*) germplasm lines/varieties.

Variety/genotype	Ecotype	Pedigree	Grain type
*Lalat*	*Indica*	OBS 677 × IR 2071 × Vikram × W1263	Long slender
*Shalimar Rice-2*	*Indica*	VL Dhan 221 × K 39	Long bold
*Basmati-1509*	*Indica*	Pusa 1121 × Pusa 1301	Long slender
*China-1007*	*Indica*	Introduction	Medium slender
*Chenab*	*Indica*	K-21/IR-2053	Medium slender
*Jhelum*	*Indica*	JAKKOKU × IET-1444	Long bold
*Koshikari*	*Japonica*	Norin-22 × Norin-1	Short bold
*SKUA-402*	*Japonica*	Kohsar × PS 86014-TR 891-7-2-1	Short bold
*K-332*	*Japonica*	Shenei × Norin-11	Short bold

**Table 2:** Apparent amylose content, dietary fiber and textural parameters of different rice genotypes.

Parameters	*Lalat*	*SR-2*	*Basmati-1509*	*China-1007*	*Chenab*	*Jhelum*	*Koshikari*	*SKUA-402*	*K-332*
Apparent amylose content (%)	28.31^a^ ± 0.50	22.24^d^ ± 1.6	23.52^b^ ± 0.6	21.46^e^ ± 0.35	22.62^c^ ± 0.20	21.0^f^ ± 0.46	15.40^i^ ± 0.48	18.22^ g^ ± 0.60	16.30^ h^ ± 0.52
Amylose class	High	Intermediate	Intermediate	Intermediate	Intermediate	Intermediate	Low	Low	Low
Dietary fiber (%)	3.97^a^ ± 0.02	2.63^f^ ± 0.03	3.15^c^ ± 0.03	2.40^g^ ± 0.05	2.78^e^ ± 0.02	2.65^f^ ± 0.02	3.45^b^ ± 0.04	2.90^d^ ± 0.04	2.96^d^ ± 0.03
**Texture profile analysis**									
Hardness (N)	4.521^a^ ± 0.04	3.382^e^ ± 0.03	3.811^b^ ± 0.06	3.295^f^ ± 0.012	3.491^d^ ± 0.04	3.262^g^ ± 0.015	3.626^c^ ± 0.03	1.979^h^ ± 0.023	1.234^i^ ± 0.016
Cohesiveness	0.794^a^ ± 0.15	0.615^e^ ± 0.15	0.762^b^ ± 0.24	0.581^f^ ± 0.07	0.623^d^ ± 0.03	0.557^g^ ± 0.37	0.683^c^ ± 0.04	0.386^h^ ± 0.15	0.312^i^ ± 0.02
Stickiness (N)	3.218^ h^ ± 0.06	3.838^e^ ± 0.05	3.640^ g^ ± 0.04	3.940^d^ ± 0.17	3.748^f^ ± 0.03	3.925^d^ ± 0.02	5.858^c^ ± 0.60	6.083^b^ ± 0.033	6.710^a^ ± 0.55

Results are expressed as mean ± SD of three replications. Mean values in the rows with different superscripts are significantly different at p ≤ 0.05.

**Table 3:** Correlation matrix between various chemical and textural parameters.

	AAC	(CT)n	RDS	SDS	RS	DF	Fat	Protein	pGI	GL	H	COH	Stickiness
AAC	1												
(CT)n	− 0.909**	1											
RDS	− 0.525	0.530	1										
SDS	0.444	− 0.446	− 0.899**	1									
RS	0.719*	− 0.755*	− 0.937**	0.847**	1								
DF	0.284	− 0.297	− 0.928**	0.880**	0.797*	1							
Fat	0.235	− 0.218	− 0.831**	0.673*	0.707*	0.771*	1						
Protein	− 0.151	− 0.084	− 0.480	0.291	0.376	0.629	0.528	1					
pGI	− 0.728*	0.827**	0.853**	− 0.801**	− 0.949**	− 0.723*	− 0.783*	− 0.690*	1				
GL	− 0.646	0.761*	0.885**	− 0.855**	− 0.944**	− 0.791*	− 0.796*	− 0.686*	0.986**	1			
H	0.729*	− 0.881**	− 0.552	0.425	0.745*	0.410	0.279	0.387	− 0.875**	− 0.818**	1		
COH	0.726*	− 0.910**	− 0.607	0.536	0.766*	0.420	0.360	0.315	− 0.900**	− 0.870**	0.924**	1	
Stickiness	− 0.893**	0.935**	0.250	− 0.166	− 0.525	− 0.014	0.029	0.099	0.639	0.540	− 0.832**	− 0.799**	1

*AAC* apparent amylose content, *(CT)n* cytosine and thymine repeats, *RDS* rapidly digestible starch, *SDS* slowly digestible starch, *RS* resistant starch, *DF* dietary fiber, *pGI* predicted glycemic index, *GL* glycemic load, *H* hardness, *COH* cohesiveness.

**Correlation is significant at the level 0.01.

*Correlation is significant at the level 0.05.

### In-vitro starch digestibility and dietary fiber

Rapidly digestible starch (RDS), slowly digestible starch (SDS), and resistant starch (RS), affect the rate of starch hydrolyzation and glucose release. RDS contribute to quick glycemic response and SDS contribute to extended glycemic response, while as RS restrains the glucose release and thus minimizes the glycemic response^32^. RDS, SDS, and RS contents showed significant variation among the tested rice varieties (Table 4). RS content of different varieties varied from 0.43 (*K-332*) to 2.85% (*Lalat*), SDS from 14.93 (*Jhelum*) to 34.72% (*Lalat*) (Table 4) and RDS from 62.43 (*Lalat*) to 84.61% (*Jhelum*). Out of all the selected varieties, *Lalat* showed lowest RDS and maximum RS and SDS followed by *Basmati-1509* and *Koshikari*. Table 3 indicated that AAC exhibited significant positive correlation (r = 0.719, p < 0.05) with RS. Many studies in past have also reported positive correlations of RS and amylose content^33–35^. It is well documented that RS content increases in cooked rice during cooling due to retrogradation^36^. The polymeric structures of amylose molecules form double helix crystals which are more resistant to zymolysis^37^. The intermediate to high amylose rice varieties usually have higher RS content after cooking as compared to low amylose varieties^15^. In present study also, RS content was recorded maximum (2.85%) in high amylose rice variety (*Lalat*) followed by *Basmati-1509* (1.85%)—an intermediate amylose variety, while as low amylose varieties, *SKUA-402* and *K-332* had relatively lower RS contents of 0.54 and 0.43%, respectively (Table 4). Similar trends were reported by Park et al.^37^ for Korean rice cultivars with different amylose contents and Chen et al.^30^ for low, intermediate and high amylose rice varieties of United States. However, *Koshikari* despite being a low amylose variety also showed higher RS content (1.17%) (Table 4), which can be attributed to its relatively higher fat (0.86%) (Table S1) and dietary fiber (DF) content (3.45%) (Table 2). Table 3 also indicated significant positive correlations of RS with fat (r = 0.707, p < 0.05). Besides RS, fat content also showed significant positive correlation with SDS (r = 0.673, p < 0.05) and negative correlation with RDS (r = − 0.831, p < 0.01) Since, interaction of amylose content and lipid fraction is responsible for formation of type-5 RS. Thus, the inference drawn was that it is not only the amylose content but its interaction with lipids which attenuates the glycemic response. The correlation studies given in Table 3 also indicated that RS is more significantly correlated with pGI (r = − 0.949, p < 0.01) as compared to AAC. Both RS and DF act in a complementary way in lowering the glycemic response^15,38^. Within the digestive tract, both RS and DF are more resistant to digestion and thus, help in lowering the blood glucose levels. In the present study, DF showed significant positive correlations with RS (r = 0.797, p < 0.05), and SDS (r = 0.880, p < 0.01) whereas, RDS showed significant negative correlation with DF (r = 0.928, p < 0.01) (Table 3).

**Table 4:** Variation in rapidly digestible starch (RDS), slowly digestible starch (SDS), resistant starch (RS) and total starch (TS) of different rice varieties.

Variety	RDS %	SDS %	RS %	TS %
*SR-2*	80.15^d^ ± 0.5	19.08^e^ ± 0.2	0.77^c^ ± 0.2	74.42^c^ ± 1.1
*Basmati-1509*	73.45^b^ ± 0.7	24.70^g^ ± 1.2	1.85^g^ ± 0.9	75.10^d^ ± 0.9
*China-1007*	83.17^f^ ± 0.4	15.88^b^ ± 0.9	0.95^e^ ± 0.7	76.15^f^ ± 1.2
*Chenab*	83.05^f^ ± 0.3	16.15^c^ ± 0.6	0.80^d^ ± 0.6	80.39^g^ ± 0.8
*Jhelum*	84.61^g^ ± 1.1	14.93^a^ ± 0.2	0.46^a^ ± 0.3	75.65^e^ ± 1.3
*Koshikari*	75.25^c^ ± 0.3	23.58^f^ ± 0.1	1.17^f^ ± 0.5	73.54^b^ ± 1.2
*SKUA-402*	81.14^e^ ± 1.4	18.32^d^ ± 0.3	0.54^b^ ± 0.3	75.77^e^ ± 1.5
*K-332*	80.10^d^ ± 0.5	19.47^e^ ± 0.5	0.43^a^ ± 0.2	76.69^f^ ± 0.7
*Lalat*	62.43^a^ ± 0.2	34.72^h^ ± 0.6	2.85^h^ ± 1.2	68.13^a^ ± 0.8

Mean values within the columns with different superscripts differ significantly at p < 0.05.

### Predicted glycemic index and glycemic load

**Figure 2:**
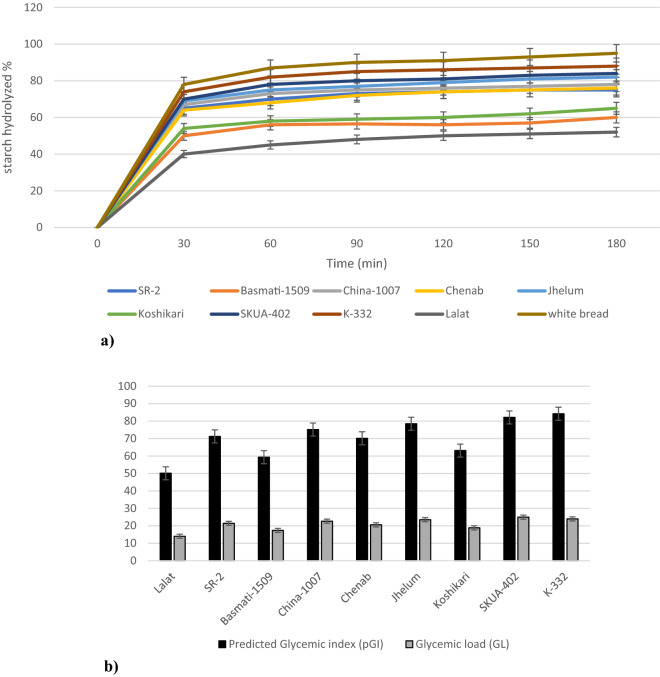
(**a**) Kinetics of starch hydrolysis of rice varieties from 0 to 180 min. (**b**) Predicted glycemic index and glycemic load of different rice varieties.

Kinetics of starch digestion analyzed for 0–180 min given in Fig. 2a revealed a rapid increase in the digestibility of starch during first 30 min of incubation followed by a steady digestion rate with the progression of time. The predicted GI values of different rice varieties ranged from 50.12 (*Lalat*) to 84.22 (*K-332*) (Fig. 2b). As per WHO^39^ classification, *SR-2, China-1007, Chenab, Jhelum, SKUA-402* and *K-332* were classified as high GI varieties (GI ≥ 70); *Basmati-1509* and *Koshikari* as medium GI varieties (GI = 56–69) and *Lalat* as low GI variety (GI ≤ 55), All the medium slender, long bold and short bold varieties elicited high GI scores which is in accordance with the results reported by Prasad et al.^15^ for popular Indian rice varieties. Although being a short bold variety, *Koshikari* showed intermediate pGI score of 63.11, which can be attributed to its higher RS, DF, fat and protein contents as compared to other short bold varieties (Table S1 and Table 2). Amylose fraction of starch in combination with lipid fraction forms type-5 resistant starch^40^. In addition, interaction between starch, lipids and proteins result in the formation of highly ordered ternary complexes with greater resistance to amylolysis^41^. Such type of complexations imparts steric hinderance to the carbolytic enzyme action and are responsible for lowering of glycemic index due to their reduced digestibility^40^. pGI exhibited significant negative correlations with fat (r = − 0.763, p < 0.05) and protein (r = − 0.690, p < 0.05). The two long slender type varieties (*Basmati-1509* and *Lalat*) selected in the present study exhibited intermediate and low GI score respectively. Ranawana et al.^42^ also reported lower glycemic scores for long-grain rice varieties. pGI showed inverse relationship with AAC (r = − 0.728, p < 0.05) and a direct relationship with (CT) repeats (r =  + 0.827, p < 0.01) (Table 3). Therefore, the low GI score (50.12) of *Lalat* can be attributed to its high AAC (28.31%) and least CT value (10) as compared to other varieties (Table 2 and Fig. 1). Fitzgerald et al.^12^ also reported strong negative correlation between amylose content, and GI in different rice varieties of Asian region. Besides AAC, RS and DF also showed negative correlations with pGI (r = − 0.949, p < 0.01; r = − 0.723, p < 0.05, respectively) (Table 3). *Lalat*, *Basmati-1509* and *Koshikari* having higher RS and DF contents showed lower glucose release after 120 min of hydrolysis which explains their GI lowering effect relative to other varieties (Fig. 2a,b). Further, the longer amylopectin chains usually present in high and intermediate amylose rice tends to form more rigid double helixes which are susceptible to retrogradation and resistant to enzymatic hydrolysis^43^. Thus, various factors other than amylose contribute to GI variation within the same amylose class in rice. Fitzgerald et al.^12^ also reported GI range of 48–92 in a diverse set of rice varieties. Chandel et al.^38^ analyzed the in-vitro GI of five rice cultivars of Chhattisgarh region and reported GI in the range of 55–68 for tested rice genotypes.

The glycemic response of food upon ingestion depends not only on the GI but also on the total amount of carbohydrates consumed. Thus, in order to estimate the glycemic response of a specific portion of food, glycemic load (GL) was also estimated. GL score of < 10 is considered as low; 11–19 as intermediate and > 20 as high^44^. For the selected set of rice varieties, GL showed significant variation between 14.04 *(Lalat*) to 24.95 (*SKUA-402*) (Fig. 2b). Out of all the selected varieties, *Lalat* (14.04), *Koshikari* (18.84) and *Basmati-1509* (17.36) were identified as intermediate GL varieties, while as *SR-2* (21.43), *China-1007* (22.64), *Chenab* (20.56), *Jhelum* (23.50), *SKUA-402* (24.95) and *K-332* (23.95) as high GL varieties. GL showed highly significant positive correlation (r =  + 0.986, p < 0.01) with pGI and negative correlations with RS (r = − 0.944, p < 0.01), DF (r = − 0.791, p < 0.05), protein (r = − 0.676, p < 0.05) and fat (r = − 0.736, p < 0.05) (Table 3). *Lalat* despite having low GI showed intermediate GL, possibly due to higher portion size selected for estimation of GL. Keeping in view, the rice consumption pattern of Northern Himalayan regions, the portion size of 100 g was chosen in the present study.

### Texture profile analysis

Textural properties of selected rice varieties determined after cooking are illustrated in Table 2. Hardness (H) and cohesiveness (COH) exhibited significant positive correlations with AAC (r = 0.729, p < 0.05; r = 0.726, p < 0.05, respectively) while stickiness showed significant negative correlation (r = − 0.893, p < 0.01) with AAC (Table 3). Stickiness of rice is governed by leaching of amylopectin over amylose, because the size of leached amylopectin molecules is many times smaller than leached amylose molecules^45^. During cooking, the amylopectin molecules leach out more in low amylose varieties, which increases their sticky behaviour and reduces their hardness and cohesiveness as compared to intermediate and high amylose rice varieties^45^. Amongst the selected Indica and Japonica varieties, the trends recorded for H and COH were *Lalat* > *Basmati-1509* > *Chenab* > *SR-2* > *China-1007* > *Jhelum* and *Koshikari* > *SKUA-402* > *K-332*, respectively, while for stickiness, the trends recorded for Indica and Japonica varieties were *Jhelum* > *China-1007* > *SR-2* > *Chenab* > *Basmati-1509* > *Lalat* and *K-332* > *SKUA-402* > *Koshikari,* respectively (Table 2). Perusal of the results given in Table 2 indicated that both H and COH were higher, while stickiness was lower in Indica varieties than Japonica ones, which is in concomitance with the previous findings reported by Lu et al. ^46^ and Li et al.^47^. Within the Indica and Japonica classes, both H and COH were higher while stickiness was lower in varieties having higher AAC (Table 2). However, *Koshikari* despite being a low amylose Japonica variety showed significantly higher hardness and cohesiveness as compared to other Japonica varieties and some intermediate Indica varieties which might be due to its higher RS and protein contents (Table 2 and Table S1). Lu et al.^46^ also reported that amount and chemical nature of solids affect the textural profile parameters of cooked rice. Apart from AAC, H and COH showed significant positive correlations with RS (r = 0.745, p < 0.05; r = 0.766, p < 0.05, respectively) (Table 3). Out of all the selected set of varieties, maximum hardness (4.515 N) and cohesiveness (0.798) were recorded in *Lalat* followed by *Basmati-1509* (H = 3.812 N and COH = 0.760) and *Koshikari* (H = 3.524 N and COH = 0.680) (Table 2). It was thus, presumed that amylose content, resistant starch and other grain components have a strong influence on textural properties of cooked rice.

## Conclusion

In the present study, the in-vitro starch digestibility and kinetics of starch digestion were studied in different rice varieties commonly cultivated and consumed in Northern Highland Himalayan regions and it was confirmed that substantial differences exist in their starch digestibility and glycemic response. Within same amylose class, significant disparity existed in glycemic index and glycemic load. It might be assumed that besides amylose content, resistant starch and other grain components like dietary fiber, protein and fat also effect the glycemic response. Although the variability within *Wx* allele at RM190 locus showed high association with the amylose content. However, the possibility that other genes may also affect the GI, cannot be precluded. Therefore, future studies are needed to identify such genes and to evaluate their effects on GI. In the present study, it was also observed that varieties having lower pGI and relatively higher RS content were harder and less sticky upon cooking. Therefore, besides enhancing the RS content and lowering the pGI, there is need to emphasis on development of soft textured high amylose rice in future rice breeding programmes related to management of diabetes. In addition to low GI rice variety-*Lalat; Basmati-1509* and *Koshikari* due to their medium pGI and relatively high resistant starch, dietary fiber and protein content can also emerge as novel varieties in future, which can be explored in long term public health strategies for management of diabetes in Northern Highland Himalayan regions, where rice is a stable food.

## Supplementary Information

Supplementary Figure S1.

Supplementary Table S1.
