# Supplementary figures and images for: Variability in waxy (Wx) allele, in-vitro starch digestibility, glycemic response and textural behaviour of popular Northern Himalayan rice varieties
Source: Sci Rep. 2021 Jun 8;11:12047. doi: 10.1038/s41598-021-91537-0 (PMC8187641; doi:10.1038/s41598-021-91537-0)

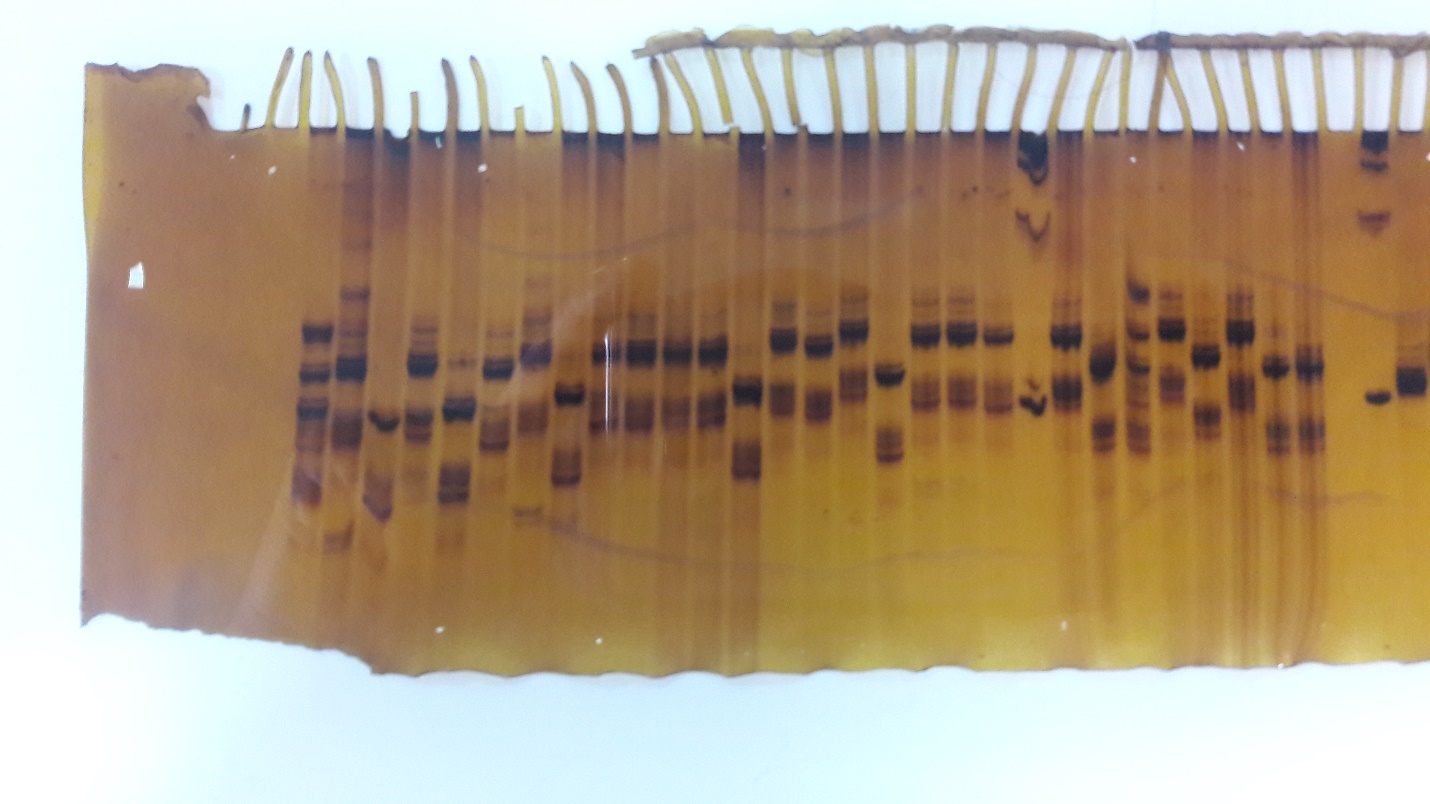


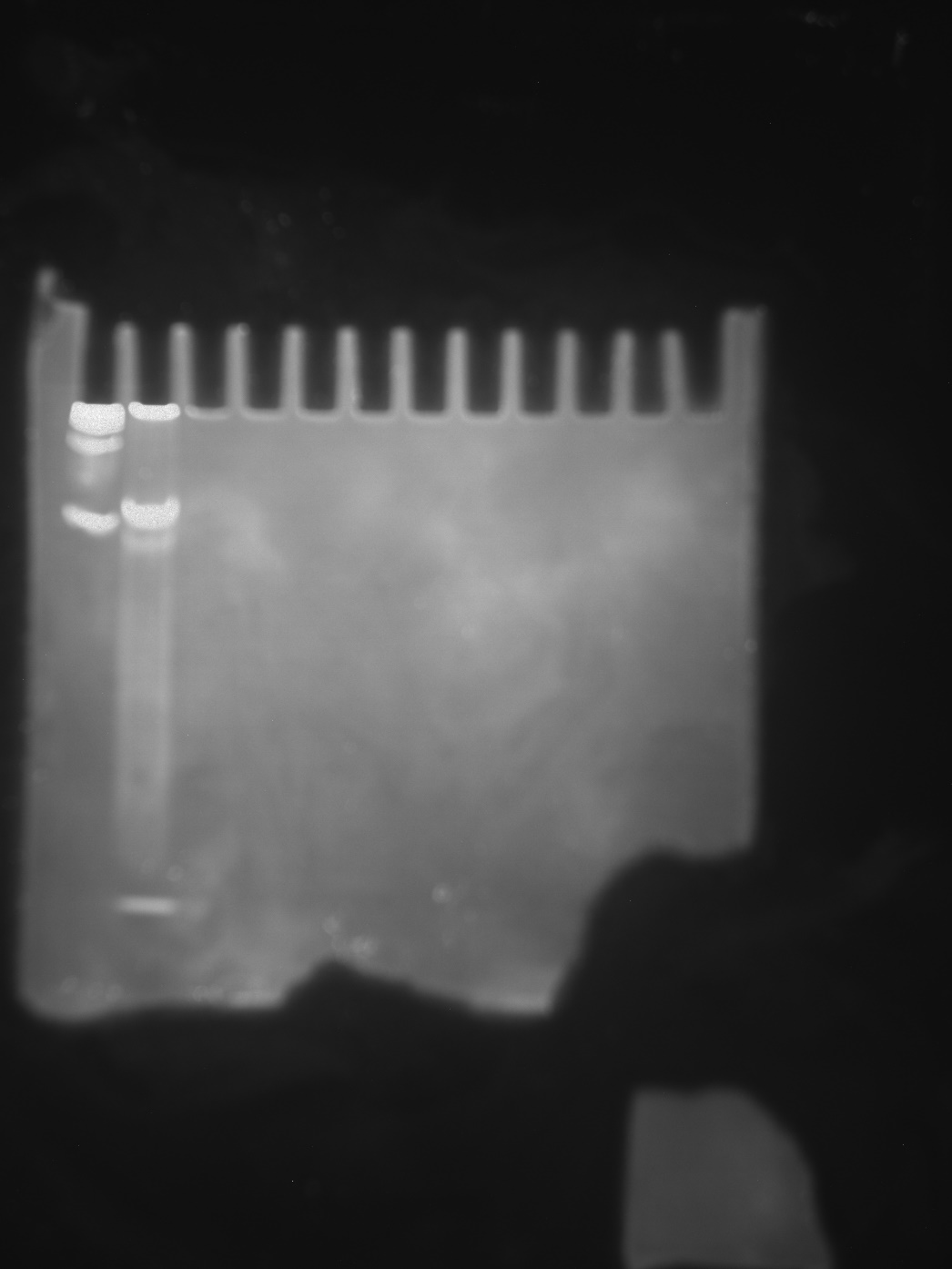


Fig. S1 Raw gel images of Figure 1

Supplement: Supplementary file 1 — Supplementary Figure S1. [file 41598_2021_91537_MOESM1_ESM.docx]
