# Supplementary material for: Variability in waxy (Wx) allele, in-vitro starch digestibility, glycemic response and textural behaviour of popular Northern Himalayan rice varieties
Source: Sci Rep. 2021 Jun 8;11:12047. doi: 10.1038/s41598-021-91537-0 (PMC8187641; doi:10.1038/s41598-021-91537-0)
Supplement: Supplementary file 2 — Supplementary Table S1. [file 41598_2021_91537_MOESM2_ESM.docx]

**Table S1 Chemical composition of selected rice varieties**

| **Chemical composition** | **Indica varieties** | | | | | | **Japonica** | | |
| --- | --- | --- | --- | --- | --- | --- | --- | --- | --- |
|  | ***SR-2*** | ***Basmati- 1509*** | ***China-1007*** | ***Chenab*** | ***Jhelum*** | ***Lalat*** | ***Koshikari*** | ***SKUA-402*** | ***K-332*** |
| Crude fat (%) | 0.59^bc^ ±0.03 | 0.53^cd^ ±0.03 | 0.66^b^±0.05 | 0.33^e^±0.06 | 0.29^e^±0.05 | 1.25^f^ ± 0.15 | 0.86^a^±0.03 | 0.45^d^±0.04 | 0.79^a^±0.07 |
| Crude protein (%) | 9.21^ac^±0.41 | 9.43^acd^±0.44 | 9.30^ac^±0.7 | 9.03^ac^±0.55 | 9.95^cd^±0.38 | 10.15^bd^± 0.33 | 10.9^b^±0.32 | 9.11^a^±0.31 | 9.4^acd^±0.5 |
| Ash (%) | 0.40^cd^±0.05 | 0.62^a^±0.05 | 0.47^bc^±0.07 | 0.44^bcd^±0.04 | 0.49^bf^±0.05 | 0.56^af^ ± 0.10 | 0.60^a^±0.04 | 0.38^ed^±0.05 | 0.30^e^±0.04 |
| Crude fiber (%) | 0.75^ab^±0.07 | 0.65^bc^±0.04 | 0.49^d^±0.05 | 0.78^a^±0.05 | 0.50^d^±0.05 | 1.09^e^ ± 0.14 | 0.45^d^±0.03 | 0.70^abc^±0.06 | 0.61^c^±0.09 |

Mean values in the rows with different superscripts are significantly different at p ≤ 0.05.
